# Haplotype Threading Using the Positional Burrows-Wheeler Transform

**DOI:** 10.4230/LIPIcs.WABI.2022.4

**Published:** 2022-08-26

**Authors:** Ahsan Sanaullah, Degui Zhi, Shaoije Zhang

**Affiliations:** Department of Computer Science, University of Central Florida, Orlando, FL, USA; School of Biomedical Informatics, University of Texas Health Science Center, Houston, TX, USA; Department of Computer Science, University of Central Florida, Orlando, FL, USA

**Keywords:** Substring Cover, PBWT, Haplotype Threading, Haplotype Matching, Applied computing → Computational biology, Applied computing → Genetics

## Abstract

In the classic model of population genetics, one haplotype (query) is considered as a mosaic copy of segments from a number of haplotypes in a panel, or threading the haplotype through the panel. The Li and Stephens model parameterized this problem using a hidden Markov model (HMM). However, HMM algorithms are linear to the sample size, and can be very expensive for biobank-scale panels. Here, we formulate the haplotype threading problem as the Minimal Positional Substring Cover problem, where a query is represented by a mosaic of a minimal number of substring matches from the panel. We show that this problem can be solved by a sequential set of greedy set maximal matches. Moreover, the solution space can be bounded by the left-most and the right-most solutions by the greedy approach. Based on these results, we formulate and solve several variations of this problem. Although our results are yet to be generalized to the cases with mismatches, they offer a theoretical framework for designing methods for genotype imputation and haplotype phasing.

## 1 Introduction

In modeling a panel of haplotype sequences arising from population genetics processes, a useful model is to view a haplotype as a mosaic copy of subsequences of other haplotypes in a panel. In other words, a haplotype (the query) is threaded through different haplotypes (as templates) in the panel. The classic Li and Stephens model parameterized this process as a hidden Markov model which can take into account the uncertainties regarding mismatches (emission probabilities) and template switching (transition probabilities) [3]. This model is quite sufficient for moderate sample sizes (hundreds to thousands) as it scales linearly with sample size. As a result, this model has served as a foundation for haplotype phasing and genotype imputation for the past two decades. However, in the biobank era when the panel size is large, the standard Li and Stephens model may not be efficient enough.

PBWT as a foundational data structure enables efficient substring matching in a population panel with aligned haplotypes [2]. For a panel with M haplotypes of length N, existing algorithms include space-efficient scanning algorithms for all-vs-all within-panel long and set maximal matches in O(MN+c) time, where c is the number of matches outputted [2]. Algorithms for time-efficient one-vs-all out-of-panel query long and set maximal match search in O(N+c) time are also available [6, 10]. These algorithms allow efficient match query on biobank scale databases.

Indeed, the PBWT has been leveraged for speeding up the Li and Stephens model. Lunter proposed a representation of the PBWT using the BWT [5]. On this representation, they perform a search for a maximum likelihood Viterbi path through the Li and Stephens Hidden Markov Model. While their algorithm can compute the optimal score in O(N) time, outputting of the haplotype threading requires O(NlogM) time. Rubinacci et al. use the PBWT to select closely related individuals efficiently. These closely related individuals are then imputed using IMPUTE5, an imputation based on the Li and Stephens model [9]. Loh et al. use the PBWT similarly for EAGLE2, their phasing algorithm. They use the PBWT to obtain representative data of a haplotype and then thread it using a haplotype copying model similar to the Li and Stephens model [4]. Lastly, Delanueau et al. use the PBWT to speed up phasing through the Li and Stephens model among other methodologies in their phasing method SHAPEIT4 [1].

However, these algorithms [1, 4, 5, 9] are designed within the Li and Stephens HMM framework and the PBWT is used as a subroutine. These algorithms mostly focus on the Viterbi path that gives the maximum likelihood solution. We argue that in the biobank-scale panel, a query may have a large number of high-quality matches, and thus outputting the single best Viterbi solution may not be informative to reveal the overall high probability possible paths. In this work, we formulate the haplotype threading problem as a combinatorial optimization problem: given a set of haplotypes X and a query z, represent z as segments of haplotypes in X and optimize a certain objective scoring function.

There are a number of possible scoring functions for threading, however there are common themes between them. Usually, one wants to represent z using a small amount of haplotypes in X or a small amount of distinct segments. In this paper, we minimize the number of segments we use to represent z. We formulate the Minimum Positional Substring Cover problem (MPSC), given a query z and a set of strings X, find a smallest set of positional substrings contained in z and a string in X that cover all characters of z. While this formulation simplifies the original haplotype threading by ignoring potential mismatches in the Li and Stephens model flavor, it enables efficient enumeration of all possible solutions, leveraging the structure of PBWT. Augmenting our algorithms with mismatch-tolerating methods such as random projection [7] or PBWT-smoothing [11], our formulation can capture the bulk of the high-probability threading paths, and thus provide flexibility for designing variations of downstream tasks such as genotype imputation and haplotype phasing.

We provide a solution in time linear to the length of the query string given a PBWT of X. Occasionally, the segment being present in only one string in X is not enough evidence to use it to represent z. Therefore, we formulate the h-Minimal Positional Substring Cover (h-MPSC) problem. Given a query string z and a set of strings X, find a smallest set of segments present in z and h strings in X. We provide a solution in O(h|𝒞|+N) time, where |𝒞| is the number of segments outputted and N is the length of z.

In Section 3, we formulate the Minimal Positional Substring Cover problem, discuss multi-allelic PBWTs, discuss properties of MPSCs, and provide a solution given a PBWT of X. In Section 4, we discuss three variations of the MPSC problem: the leftmost, rightmost, and set maximal match only minimal positional substring covers. Then we show that our original solution is leftmost and provide linear time solutions for the rightmost and set maximal match only MPSC problems. In Section 5, we discuss the h-MPSC problem and provide a solution in O(h|𝒞|+N) time. Then, in Section 6 we discuss boundary cases in these algorithms. Lastly, we cover possible future work in haplotype threading related to the Minimal Positional Substring Cover problem in Section 7.

## 2 Background

We index the characters of a string z with N characters from 0 to N-1. The first character is z[0] and the last character is z[N-1]. The string z[i,j) is the substring of z that starts at character i and ends at character j-1.z[i,j] is the substring of z that starts at character i and ends at character j.z(i,j] is the substring of z that starts at character i+1 and ends at character j,z(i-1,j]=z[i,j]=z[i,j+1).

**A positional substring** of a string z is a 3-tuple, (i,j,z), where i and j are nonnegative integers, i≤j+1≤|z|, and z is the “source” of the substring. The substring corresponding to the positional substring (i,j,z) is z[i,j]. If i=j+1, then (i,j,z) corresponds to the empty string, ε. The number of characters in z is j-i+1. A non-empty positional substring (i,j,z) is contained (or present) in a string s if 0≤i≤j<|s| and s[i,j]=z[i,j]. A positional substring (i,j,z) corresponding to an empty string is contained in a string s iff 0≤j≤|s|. Two positional substrings, (i,j,s) and (k,l,t) are equal iff i=k,j=l, and s[i,j]=t[k,l].

**A positional substring cover 𝒞** of a string z by a set of strings X is a set of positional substrings such that every character of z is contained in a positional substring and every positional substring in the set is present in z and a string in X. I.E., ∀k∈{0,…,|z|-1},∃(i,j,s)∈𝒞 s.t. i≤k≤j, and ∀(i,j,s)∈𝒞,∃x∈X s.t. x[i,j]=z[i,j]=s[i,j]. The “source” s of a positional substring (i,j,s) in a positional substring cover can be any string s s.t. s[i,j]=x[i,j]=z[i,j] for some x∈X. The size of a positional substring cover is the number of positional substrings it contains.

π is a projection on tuples. $\pi_{1}(i,j,z)=i,\pi_{2}(i,j,z)=j$, and $\pi_{3}(i,j,z)=z$.

### 2.1 Positional Burrows-Wheeler Transform

The Positional Burrows-Wheeler Transform (PBWT) is an index on X, a set of M binary strings of length N. It was published by Richard Durbin in 2014 [2]. It allows efficient search for positional matches in binary strings. Furthermore, it allows efficient compression of binary strings that have local correlation. The main idea of the PBWT is it stores four two-dimensional integer arrays of size around M×N. The prefix array, a contains N+1 sortings of the M binary strings. Sorting i in a contains the strings x∈X sorted by their reversed prefixes of length i. If two strings have the same reversed prefix of length i, their relative order in a[i] and a[0] are the same. The divergence array d contains N+1 integer arrays of length M. The j-th integer in the i-th array, d[i][j], contains the starting position of the longest match between a[i][j] and a[i][j-1] that ends at i. The u array has N arrays of length M. The j-th position in the i-th array contains the position the string a[i][j] would have in a[i+1] if it had a 0 at index i. The v array has N arrays of length M. The j-th position in the i-th array contains the position the string a[i][j] would have in a[i+1] if it had a 1 at index i.

We now define the PBWT with notation commonly used in this paper in order to give the reader a deeper understanding of the PBWT and this paper's notation. We use R(z) to denote the reverse of the string z. Call $y_{i,j}$ the string corresponding to the ID a[i][j]. Each binary string in X is given a unique integer ID in {0,…,M-1}. The a[i] array contains the IDs of the strings x∈X sorted by R(x[0,i)). $d[i][j]={\text{min}}_{k\in \left\{l\in \{0,\ldots ,i\}:y_{i,j}[l,i)=y_{i,j-1}[l,i)\right\}}\;k.\;u[i][0]=0$. If $y_{i,j}[i]=0,u[i][j+1]=u[i][j]+1$. Otherwise, u[i][j+1]=u[i][j]. v[i][0]=u[i][M-1]+1 if $y_{i,M-1}[i]=0$, otherwise v[i][0]=u[i][M-1]. If $y_{i,j}[i]=1,v[i][j+1]=v[i][j]+1$. Otherwise, v[i][j+1]=v[i][j]. The definitions in this paragraph and the one above are equivalent.

Richard Durbin introduced useful definitions of matches between strings. A match between two strings, s and t((i,j,s)=(i,j,t)), is **locally maximal** if it can’t be extended in any direction and still match. (s[i-1]≠t[i-1] or i=0) and (s[j+1]≠t[j+1] or j=N-1). When comparing a string s to a set of strings X, a match from s to x∈X, ((i,j,s)=(i,j,x)), is set **maximal from** s **to** X if it is locally maximal and there does not exist a match from s to a string in X that is larger and contains this match. ∀t∈X∀k∈{0,…,i}∀l∈{j,…,M}, (k=i and l=j) or t[k,l]≠s[k,l]. Lastly, a match from s to x∈X,(i,j,s)=(i,j,x), is a **longest match from** s **to** X **ending at index j** if it is a longest match between s and all strings in X that ends at index j. ∀t∈X∀k∈{0,…,i}, k=i or t[k,j]≠s[k,j].

## 3 Minimal Positional Substring Cover

**Figure 1:**
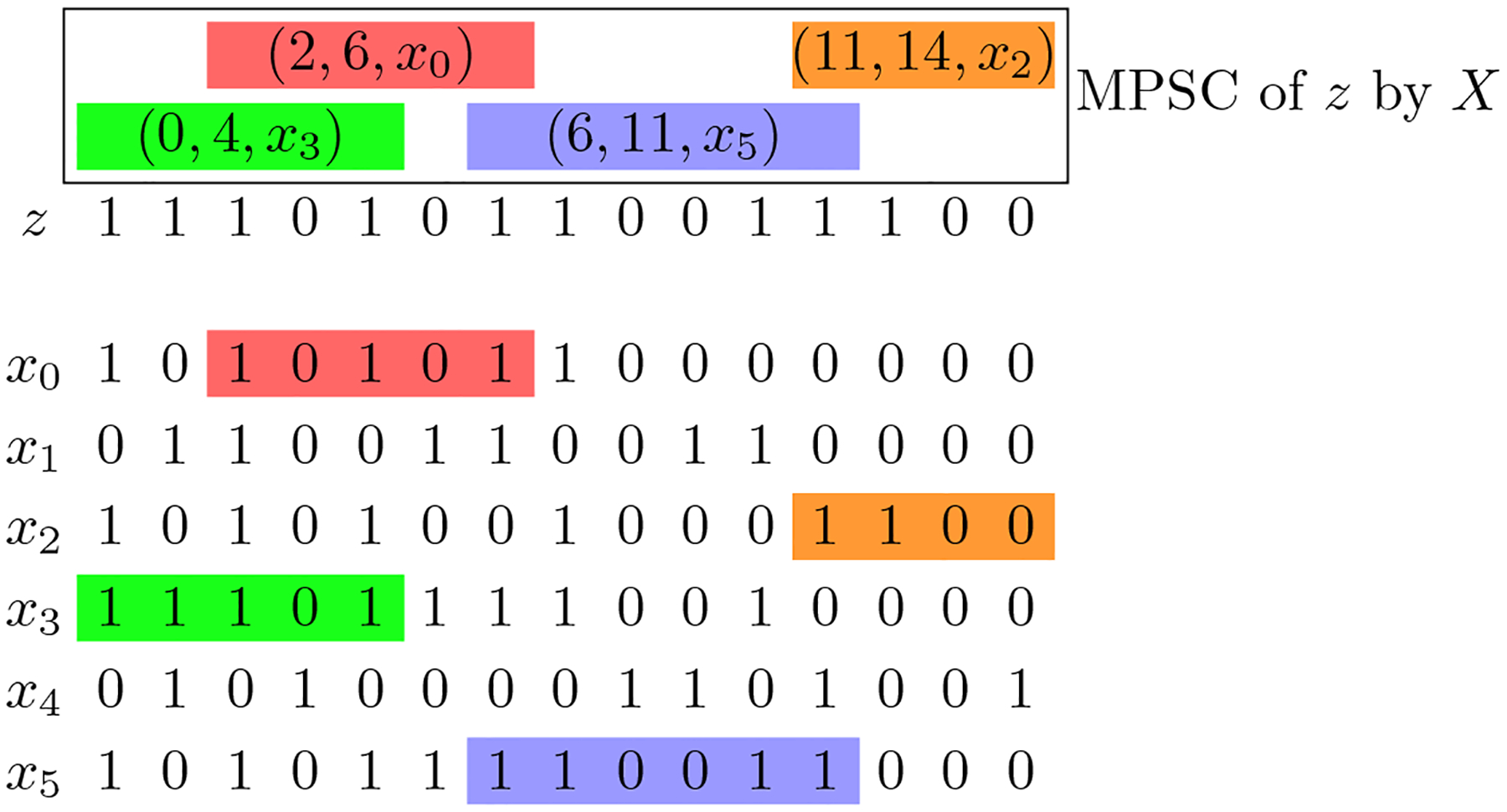
A minimal positional substring cover of z by X.

The Minimal Positional Substring Cover (MPSC) problem is, given a set X of M strings and a string z, find a positional substring cover of z by X of the smallest size out of all positional substring covers of z by X. Call this cover a minimal positional substring cover of z by X. Refer to Figure 1 for a depiction of an MPSC.

### 3.1 Properties

▷ Claim 1. A minimal positional substring cover of z by X exists if and only if for every i∈{0,…,|z|-1}, there exists a string in X that has the same character as z at index i.

Proof. If there exists an i∈{0,…,|z|-1} s.t. ∀x∈X,z[i]≠x[i], there exists no positional substring cover of z by X since a positional substring that covers index i and is contained in z and a string in X doesn’t exist. There doesn’t exist a positional substring cover of z by X, so a minimal positional substring cover of z by X doesn’t exist.

If ∀i∈{0,…,|z|-1}, ∃x∈X s.t. z[i]=x[i], then there exists a positional substring cover 𝒞 of z by X. 𝒞={(i,i,z):i∈{0,…,|z|-1}}. A positional substring cover of z by X exists, so a minimal positional substring cover of z by X exists. ◁

▷ Claim 2. For a minimal positional substring cover 𝒞 of z by X, every index k∈{0,…,|z|-1} is contained in at most two of its positional substrings. $\forall k\in \{0,\ldots ,|z|-1\},\exists \left(i_{0},i_{0},s_{0}\right)\left(i_{1},j_{1},s_{1}\right)\in 𝒞$ s.t $\forall \left(i_{2},i_{2},s_{2}\right)\in 𝒞,i_{2}<k<i_{2}⟺\left(\left(i_{2}=i_{1}\wedge j_{2}=i_{1}\wedge s_{2}=\right.\right.\left.\left.s_{1}\right)\vee \left(i_{2}=i_{0}\wedge j_{2}=j_{0}\wedge s_{2}=s_{0}\right)\right)$. Note that every index is contained in at least one positional substring by the definition of positional substring cover, therefore, every index is contained in at least one and at most two positional substrings in 𝒞.

Proof. Suppose there exists an index k that is contained in more than two positional substrings in a minimal positional substring cover 𝒞 of z by X. Take 𝒟, the set of positional substrings in 𝒞 that contain i, $𝒟=\left\{p\in 𝒞:\pi_{1}(p)\leq i\leq \pi_{2}(p)\right\}$. Take the positional substrings p,q∈𝒟 that start the earliest and end the latest respectively. $\forall r\in 𝒟,\pi_{1}(p)\leq \pi_{1}(r)$ and $\pi_{2}(q)\geq \pi_{2}(r)$. p covers at least $\left[\pi_{1}(p),i\right]$ and q covers at least $\left[i,\pi_{2}(q)\right]$. Therefore, all the indices contained in positional substrings in 𝒟 are covered by p and q. Therefore, removing all positional substrings in 𝒟 except p and q from 𝒞 would yield a smaller set of positional substrings that covers z by X (since |𝒟|>2). This contradicts the fact that 𝒞 is a minimal positional substring cover of z by X. ◁

▷ Claim 3. Given a minimal positional substring cover 𝒞 of z by X, the starting points of all positional substrings in 𝒞 are unique and their ending points are unique. $\forall p,q\in 𝒞,p\neq q⟹\left(\pi_{1}(p)\neq \pi_{1}(q)\wedge \pi_{2}(p)\neq \pi_{2}(q)\right)$.

Proof. Suppose that there are two positional substrings in 𝒞 that start at the same position, i. Then, the one with the smaller ending position can be removed from 𝒞. This new set is a smaller positional substring cover of z by X because all of the sites are still covered by positional substrings contained in z and a string in X. This contradicts the assumption that 𝒞 is a minimal positional substring cover of z by X. Similar reasoning can be applied to positional substrings with the same ending position. ◁

▷ Claim 4. For a minimal positional substring cover 𝒞 of z by X. For any positional substring p∈𝒞, if p has the i-th smallest starting point out of all positional substrings in 𝒞, it also has the i-th smallest ending point out of all positional substrings in 𝒞. $\forall p,q\in 𝒞,\pi_{1}(p)<\pi_{1}(q)⟺\pi_{2}(p)<\pi_{2}(q)$.

Proof. Suppose there are two positional substrings, p,q∈𝒞 s.t. the order of their starting points and ending points are different. Without loss of generality, say p starts earlier. Then, we have $\pi_{1}(p)<\pi_{1}(q)$ and $\pi_{2}(p)>\pi_{2}(q)$. In this case, q is completely covered by p. Removing q from 𝒞 results in a positional substring cover of z by X that is smaller than 𝒞. This contradicts the assumption that 𝒞 is a minimal positonal substring cover. ◁

We use Claim 4 to define i-th positional substring in a minimal positional substring cover of z by X. The i-th positional substring in a minimal positional substring cover 𝒞 is the positional substring with the i-th smallest starting position in the cover, 0-indexed. This is equivalent to ordering by ending position (by Claim 4). We use 𝒞[i] to denote the i-th positional substring of 𝒞.

### 3.2 Main Idea

We present a solution to the Minimal Positional Substring Cover problem here. This solution assumes that a PBWT of X is provided. Note that a PBWT specifies a binary alphabet, whereas the Minimal Positional Substring Cover problem doesn’t assume a binary alphabet. There are two ways to deal with this. Firstly, it is possible to convert a string s in an arbitrary alphabet Σ into a binary string where every character is represented by ⌈log|Σ|⌉ binary characters. Minor modifications on the PBWT algorithms would provide algorithms that take a multiplicative factor of O(log|Σ|) extra time and space. Another solution is as follows. Instead of two arrays, u, and v, that keep track of position of the sequence with 0 and 1 respectively, keep one three dimensional array w, that keeps track of position of the sequence with each character. Then, w[i][j][c]=u[i][j] in the original formulation if c=0 and v[i][j] if c=1. In general, w[i][j][c] is the position in a[i+1][j] of sequence a[i][j] if it had a c at index i. Minor modifications of query algorithms on the PBWT would run on this data structure with no extra time, however a multiplicative factor of O(|Σ|) extra space is taken. We will use the second solution for an arbitrary alphabet PBWT for the rest of this paper. The construction and all vs. all match algorithms for this second solution have been explored in a paper by Naseri et al. [8].

**Figure 2:**
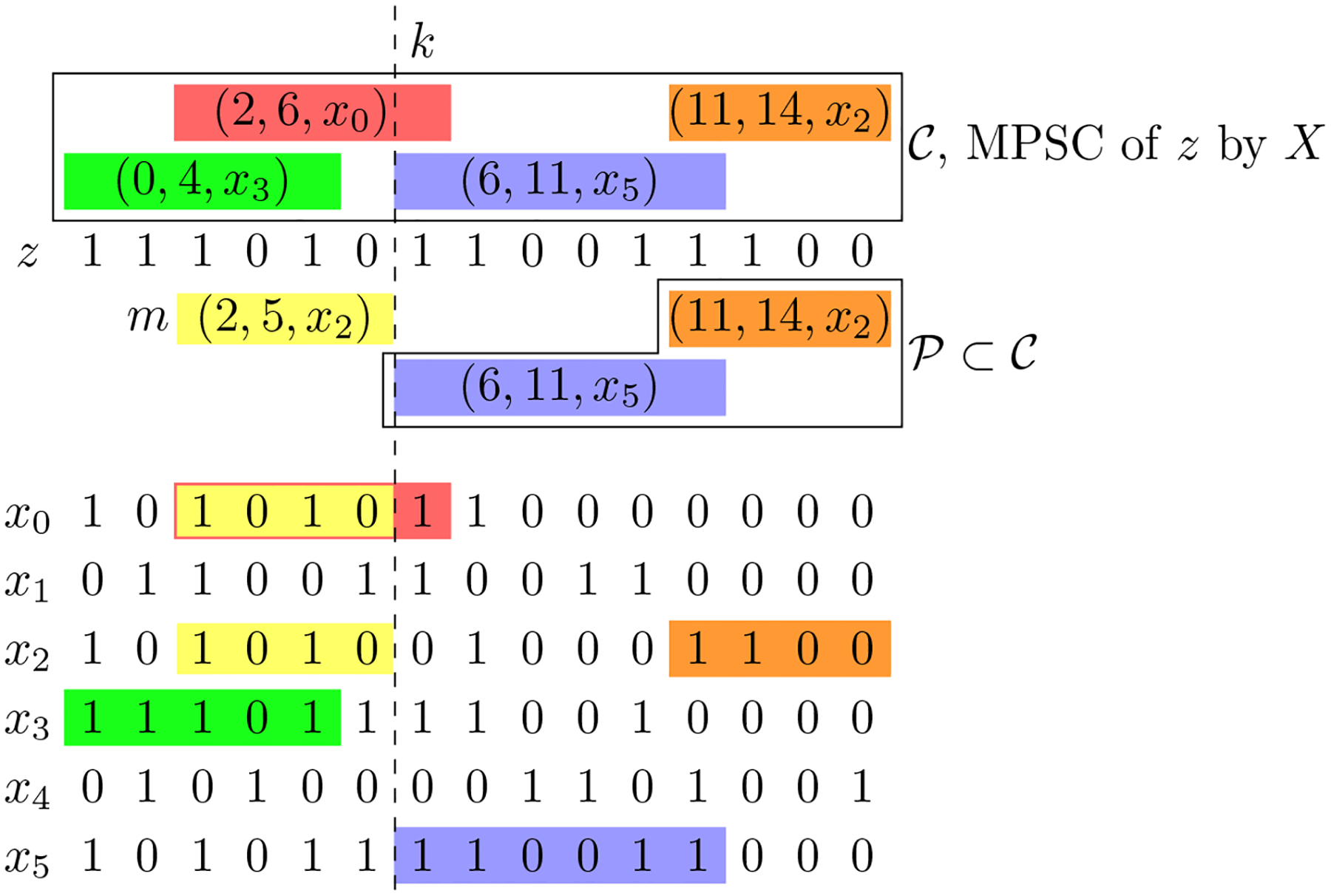
Depiction of Lemma 1. 𝒞 is a MPSC of z by X. 𝒫 is a subset of 𝒞 that covers only indices {k,…,N-1}. The longest match m ending at index k-1 and 𝒫 form a subset of a MPSC 𝒟 of z by X. 𝒫∪{m}⊆𝒟.

We start with 𝒫=∅. Add to 𝒫 the positional substring corresponding to the longest match from z to X ending at N-1. Every minimal positional substring cover of z by X must cover index N-1. Replacing the positional substring in any such cover with the longest match from z to X ending at N-1 will yield a minimal positional substring cover of z by X because the sets are the same size and the longest match ending at N-1 covers all sites any match ending at N-1 covers by definition. Now, 𝒫 is a subset of a minimal positional substring cover 𝒞 of a string z by X. It covers indices {k,…,N-1}. For some integer 0<k≤N-1. And none of the indices {0,…,k-1}. We will show in Lemma 1 that given a subset of a minimal positional substring cover that covers a contiguous section of indices, the longest match ending at the last index it doesn’t cover is also in a minimal positional substring cover. Using Lemma 1, we add the positional substring corresponding to the longest match ending at k-1 to 𝒫. We repeat this with our new 𝒫 and new k until 𝒫 is a minimal positional substring cover of z by X. Refer to Figure 2 for a depiction of Lemma 1.

**▶ Lemma 1** (Minimal Positional Substring Cover Modularity). *Given a subset* 𝒫 *of a minimal positional substring cover* 𝒞 *of a string* z *by* X *that covers all the indices in* {k,…,N-1} *and none of the indices in* {0,…,k-1} *for* 0<k≤N-1. *Take* m=(i,k-1,s), *the longest match ending at* k-1 *from* z *to* X. 𝒫∪{m} *is a subset of a minimal positional substring cover of* z *by* X.

**Proof.** If 𝒫 is a subset of a minimal positional substring cover 𝒞 of a string z by X, then, the (|𝒞|-|𝒫|-1)-th positional substring in 𝒞 must cover index k-1. If it didn’t, either the index k-1 is not covered by 𝒞, or another substring in 𝒞 covers index k-1. In the first case, our definition of 𝒞 is contradicted.

In the second case, if an n-th positional substring covers k-1, if n>|𝒞|-|𝒫|-1, our defintion of 𝒫 is contradicted. If n<|𝒞|-|𝒫|-1, our definition of n-th positional substring is contradicted (n-th positional substring ends after (|𝒞|-|𝒫|-1)-th positional substring), or 𝒞 is not a minimal positional substring cover ((|𝒞|-|𝒫|-1)-th positional substring starts after k-1, in which case it can be removed while maintaining the coverage of all sites).

Now, for any 𝒞, replacing the (|𝒞|-|𝒫|-1)-th positional substring with the longest match ending at k-1 of z and X will yield a minimal positional substring cover of z by X. This is because the new set is the same size as 𝒞 and all the sites 𝒞 covered are also covered by the new set. Any indices greater than k-1 are covered by 𝒫 and there are no indices less than k-1 that the original (|𝒞|-|𝒫|-1)-th positional substring covered that the longest match ending at k-1 doesn’t cover by the definition of longest match ending at k-1. ◀

Obtaining the longest match ending at index i is easy using the PBWT. At any index i, for some sequence a[i+1][j], the longest match ending at i between it and $X-\left\{y_{i+1,j}\right\}$ starts at the smaller of d[i+1][j] and d[i+1][j+1] (the starting position of the longest match ending at i between {a[i+1][j],a[i+1][j-1]} and {a[i+1][j],a[i+1][j+1]} respectively. Precisely, the longest match ending at i from z to X is (min(d[i+1][j],d[i+1][j+1]),i,z). Unfortunately, the query sequence is not in the PBWT, so using this method directly is not possible. In 2021, Sanaullah et al. introduced a method of “virtually inserting” a query haplotype into a PBWT in time linear with respect to the length of the haplotype [10]. This calculates the positions in the prefix array and the divergence values a query string would have if it was present in a PBWT.

### 3.3 Algorithm

A virtual insertion entails the calculation of the positions the string would take in the prefix arrays a, and the calculation of the new divergence values of the string and the string below it in every prefix array index. The original algorithm was described for binary strings. Here we describe the algorithm on an arbitrary alphabet PBWT. We begin by calculating the locations of the query z in the prefix arrays.

We will keep track of the index t[k], k∈{0,…,N}. t[k] is the index in the k-th prefix array $\left(a\left[k\right]\right)$ which the query sequence z would be placed above if it were in the PBWT. Choose t[0] arbitrarily, we choose t[0]=0. The position z would be above in a[k+1] is the position t[k] would be at in a[k+1] if it had z[k] at position k. In other words, t[k+1]=w[k][t[k]][z[k]]. We can use the w array to calculate each t[k] in constant time. Overall, O(N) where N=|z|. Next, we calculate the divergence values.

The key observation for the efficient calculation of divergence values is that the divergence value of the query sequence z at index k will be less than or equal to its divergence value at index k+1. The same holds for the divergence value of the sequence below z. See Section 2.3 of [10] for a proof of this claim. Therefore, we calculate two integer arrays, $d_{z}$ and $d_{\text{below}z}$ of size N+1. We calculate $d_{z}[N]$ and $d_{\text{belowz}}[N]$ by starting at d=N and decrementing until $z[d-1]\neq y_{N,t[N]-1}[d-1]$ for $d_{z}$ (and until $z[d-1]\neq y_{N,t[N]}[d-1]$ for $\left.d_{\text{belowz}}\right)$. Then, we set k=N-1→0 and calculate $d_{z}[k]$ and $d_{\text{belowz}}[k]$ by starting at $d_{z}[k+1]$ and $d_{\text{below}z}[k+1]$ respectively and decrementing until the next characters are not equal, see condition in previous sentence. Overall, this takes O(N) time since every index has a constant cost and over all the sites, the divergence calculation is a counter from N to 0.

As we noted before, obtaining the longest match ending at index k is easy given a PBWT of binary strings. This still holds in our arbitrary alphabet PBWT. The longest match ending at k of a[k+1][j] to $X-\left\{y_{k+1,j}\right\}$ will be adjacent to it in a[k+1], either above or below it. For our query z, the longest match ending at k starts at the smaller of $d_{z}[k+1]$ and $d_{\text{belowz}}[k+1]$. Therefore, using Lemma 1 after virtual insertion, we can output a minimal positional substring cover of z by X. Add the longest match ending at N-1(j,N-1,z) to the cover, save k=j. Repeat the following until k=0: Add the longest match ending at k-1, (j,k-1,z) to the cover, set k=j. At the end of this process, we have a minimal positional substring cover of z by X. Refer to Algorithm 1 for the pseudocode of this algorithm. Note that there are some boundary cases in the algorithm not included in the pseudocode, these are discussed in Section 6.


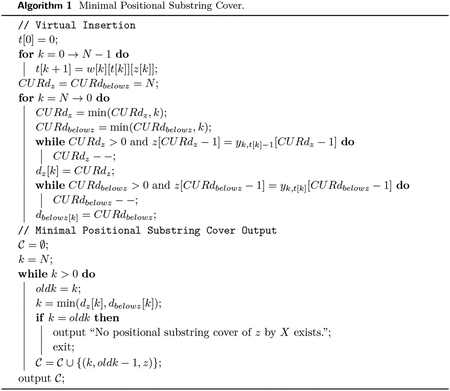


### 3.4 Time Complexity

The time complexity of this algorithm is O(N) where N is the length of the query string. The calculation of locations in the prefix array is clearly O(N) since the calculation of each index is constant time and there are N indices. The calculation of divergence values is not constant time per index, however each index incurs a constant cost and a variable cost. Over all N indices, the sum of the variable costs is O(N) since the variable costs are counters from N to 0. Therefore, the virtual insertion part of the algorithm takes O(N) time. The minimal positional substring cover output section of the algorithm takes constant time per positional substring in the ouput, therefore it takes O(|𝒞|) time. The size of a minimal substring cover of z by X is at most N because there may not be any empty positional substrings in a minimal positional substring cover. Therefore, given a PBWT of a set of M strings, and a query string z of length N, Algorithm 1 outputs a minimal positional substring cover of z by X in O(N) time.

## 4 Problem Variants

### 4.1 Leftmost Minimal Positional Substring Cover

A leftmost minimal positional substring cover 𝒞 of z by X is a minimal positional substring cover of z by X with the following property: for any i-th substring in 𝒞, it starts at least as early as the i-th substring of every other minimal positional substring cover of z by X. We use i-th positional substring of an MPSC as defined in Section 3.1. With this notation, a minimal positional substring cover 𝒞 of z by X is leftmost if ∀i∈{0,…,|𝒞|-1}∀𝒟∈{𝒫:𝒫isaminimalpositionalsubstringcoverofzbyX}, $\pi_{1}(C[i])\leq \pi_{1}(D[i])$.

▷ Claim 5. If the i-th substring in a leftmost minimal positional cover 𝒞 of z by X begins at index j, every (i-1)-th substring in a minimal positional substring cover of z by X contains index j-1. ∀i∈{1,…,|𝒞|-1}∀𝒟∈{𝒫:𝒫isaminimalpositionalsubstringcoverofzbyX}, $\pi_{1}(𝒟[i-1])\leq \pi_{1}(𝒞[i])-1\leq \pi_{2}(𝒟[i-1])$.

Proof. Suppose there existed an i∈{1,…,|𝒞|-1} and 𝒟∈{𝒫:𝒫isaminimalpositionalsubstringcoverofzbyX} such that $\pi_{1}(𝒞[i])-1>\pi_{2}(𝒟[i-1])$. Then, since 𝒟 is a cover of z by X, it contains a positional substring that contains index $\pi_{1}(𝒞[i])-1$, call it 𝒟[j]. If j<i-1, the definition of i-th positional substring is contradicted since $\pi_{2}(𝒟[j])>\pi_{2}(𝒟[i-1]$ and j<i-1. If j>i-1,j=i by definition of i-th positional substring, and 𝒞 is not leftmost since $\pi_{1}(𝒞[i])>\pi_{1}(𝒟[i])$. Therefore, no such i and 𝒟 exist.

Suppose there existed an i∈{1,…,|𝒞|-1} and 𝒟∈{𝒫:𝒫isaminimalpositionalsubstringcoverofzbyX} such that $\pi_{1}(𝒞[i])-1<\pi_{1}(𝒟[i-1])$. Then the set {𝒟[j]:0≤j<i-1} covers the indices $\left[0,\pi_{1}(𝒟[i-1])-1\right]$ with i-1 positional substrings and the set {𝒞[j]:i≤j<|𝒞|} covers the indices $\left[\pi_{1}(𝒞[i]),N-1\right]$ with |𝒞|-i positional substring. Their union is a positional substring cover of z by X with |𝒞|-1 positional substrings. This contradicts the assumption that 𝒞 is a minimal positional substring cover of z by X, therefore, no such i and 𝒟 exist. ◁

Here, we will show that the minimal positional substring cover 𝒞 of z by X outputted by Algorithm 1 is leftmost. The (|𝒞|-1)-th substring in any MPSC 𝒟 of z by X must cover index N-1. This is because it is the substring with the greatest ending position in 𝒟 (in order for 𝒟 to be minimal) and 𝒟 must cover index N-1. The leftmost starting point of any positional substring that ends at N-1 contained in z and a string in X is the starting point of the longest match from z to X ending at index N-1. This is exactly the (|𝒞|-1)-th positional substring in 𝒞.

Therefore, the set containing only the 𝒞[|𝒞|-1] is a subset of a leftmost minimal positional substring cover of z by X. By Claim 5, the (|𝒞|-2)-th substring of a leftmost minimal positional substring cover must contain index $\pi_{1}(𝒞[|𝒞|-1])-1$. The leftmost starting point of any positional substring that ends at $\pi_{1}(𝒞[|𝒞|-1])-1$ contained in z and a string in X is the longest match between z and X ending at that index. This is exactly the (|𝒞|-2)-th positional substring in 𝒞. Therefore, the set {𝒞[|𝒞|-2],𝒞[|𝒞|-1]} is a subset of a leftmost minimal positional substring cover of z by X. This logic can be repeated for every positional substring in 𝒞 to show that it is a leftmost minimal positional substring cover of z by X.

### 4.2 Rightmost Minimal Positional Substring Cover

A rightmost minimal positional substring cover 𝒞 of z by X is a minimal positional substring cover of z by X with the following property: for any i-th substrings, it ends at least as late as the i-th substring of every other minimal positional substring cover of z by X. ∀i∈{0,…,|𝒞|-1}∀𝒟∈{𝒫:𝒫isaminimalpositionalsubstringcoverofzbyX}, $\pi_{2}(𝒞[i])\geq \pi_{2}(𝒟[i])$.

The logic for obtaining a rightmost minimal positional substring cover is similar to that for a leftmost. One method is to find the leftmost cover using Algorithm 1 of the query R(z) and $X_{\text{R}}=\{\text{R}(x):x\in X\}$, then reverse all positional substrings in the outputted cover. This would require building a PBWT on $X_{\text{R}}$, which would take O(|Σ|MN) time. Another method is to build it with the longest matches starting at i. The first positional substring the cover 𝒞 would be the longest match starting at index 0. The second would be the longest match starting at $\pi_{2}(𝒞[0])+1$, the third at $\pi_{2}(𝒞[1])+1$, and so on. The proof for this cover being rightmost is very similar to the leftmost proof.

To obtain the longest match from z to X starting at index k efficiently, create an analogous array to the divergence array for longest match starting at k. Take array δ of length N where $\delta [i]=\text{min}\left(d_{z}[i+1],d_{\text{below}z}[i+1]\right)$ for i∈{0,…,N-1} (use the definitions of $d_{z}$ and $d_{\text{belowz}}$ from Algorithm 1). Create the analogous array b of length N where $b[j]={\text{max}}_{i\in \{k\in \{0,\ldots ,N-1\}:\delta [k]\leq j\}}\;i$ for j∈{0,…,N-1}. This can be done in O(N) time. Now b[j] corresponds to the end of the longest match starting at j from z to X. Therefore, we can build a rightmost minimal positional substring cover of z by X in O(N) time given a PBWT of X.

### 4.3 Minimal Positional Substring Cover Using Set Maximal Matches

In haplotype threading, it is usually better to have longer matches when possible. This is because matches that are longer are usually between individuals that are more closely related. In this case, it may be useful to have a MPSC of z by X that is composed of only set maximal matches. Here we provide a brief description of how to output a MPSC of z by X containing only set maximal matches in O(N) time given a PBWT of X.

We begin by running Algorithm 1 on z and the PBWT of X. Call its outputted cover 𝒟. All the positional substrings (i,j,s) in 𝒟 are longest matches ending at j from z to X, therefore the longest match (m) starting at i from z to X is a set maximal match from z to X. This is because for another match to encompass this match, it needs to contain the indices {i,…,j} and start earlier or end later. No match starts earlier because (i,j,s) is the longest match ending at j. No match ends later because m is the longest match starting at i. Therefore, in order to obtain an MPSC containing only set maximal matches, we just have to replace every positional substring (i,j,s)∈𝒟 with the longest match starting at i from z to X. We can do this efficiently using the b array from Section 4.2. b[i] contains the ending point of the largest match starting at i. We can compute the b array in O(N) time. We can replace every match in 𝒟 in O(1) time and |𝒟|≤N. Therefore we can obtain a MPSC composed of only set maximal matches in O(N) time.

## 5 h-Minimal Positional Substring Cover

The h-Minimal Positional Substring Cover problem, is, given a query string z, and a set of strings X, find the smallest cover out of all positional substrings covers 𝒞 of z by X where every positional substring in 𝒞 is contained in at least h strings in X. The solution to this problem is similar to the solution to the Minimal Positional Substring Cover problem, and it is biologically useful because the large group of similar individuals for every region suggests that they are closely related. Note that Claims 2–4 hold for h-MPSCs. Furthermore, with this definition, a 1-MPSC is a MPSC of z by X and a MPSC is a 1-MPSC of z by X.

The h-Minimal Positional Substring Cover problem is similar to the Minimal Positional Substring Cover problem. The main difference is that in the new problem, we only consider positional substrings that have h matches in X. Any h-MPSC will contain a positional substring that contains the last index and is contained in h strings in X by definition. We can replace this positional substring with the largest match ending at index N-1 contained in h strings in X. This will result in a set that is still a h-MPSC because it covers all the same sites as the previous set and is the same size. Therefore, we start building a h-MPSC with the longest match ending at index N-1 with h matches. Finding this match is easy using the PBWT.

### 5.1 Longest Match ending at k present in h strings in X

**Figure 3:**
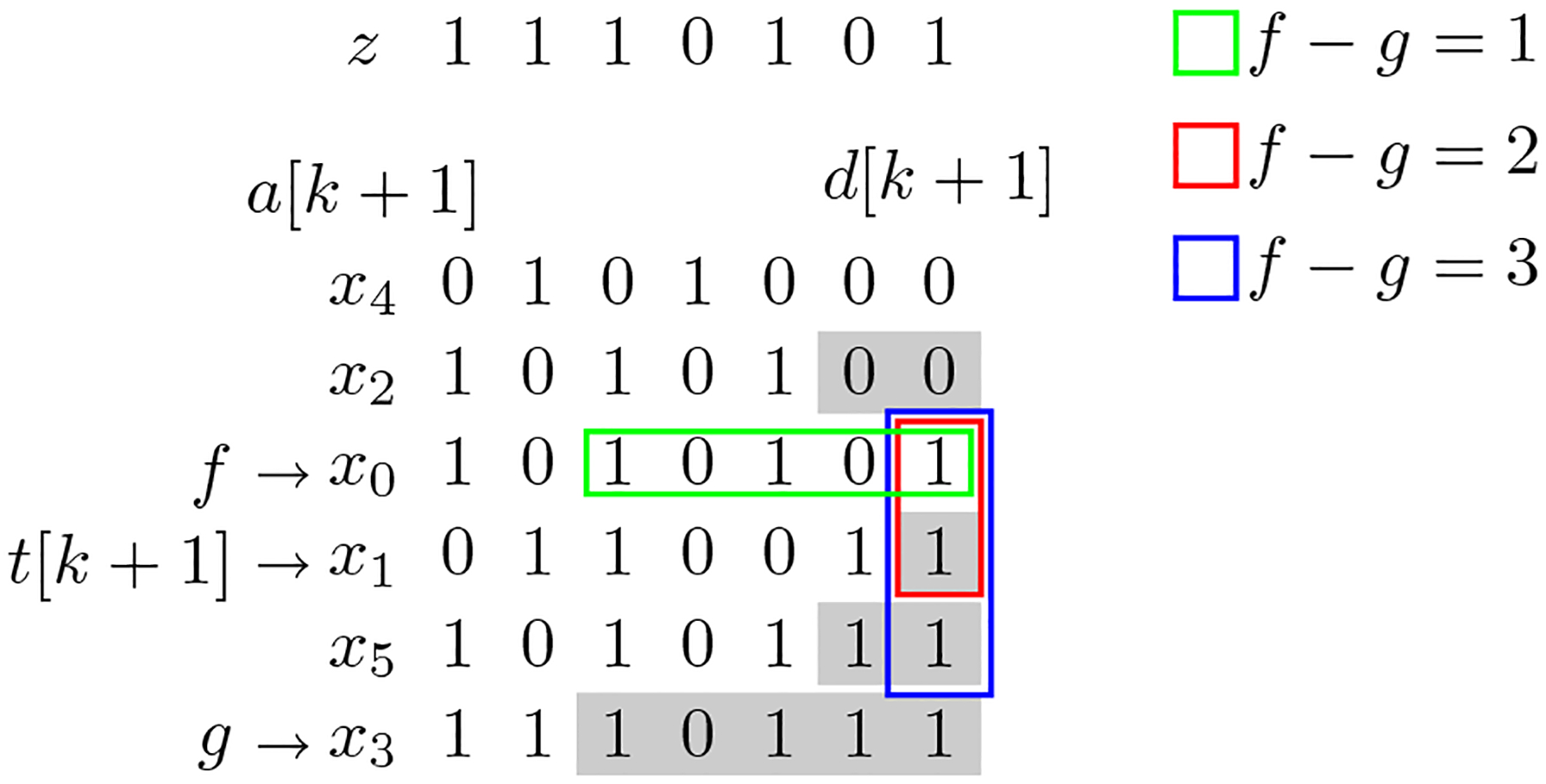
Finding the longest match ending at k present in h strings in X for k=6 and h=3. We only depict indices {0,…,k} for all strings. Highlighted in gray are divergence vales, d[k+1][j]. f and g values depicted are the final f and g values. The window is depicted for f-g∈{1,2,3}.

Once a query string z is virtually inserted into a PBWT, it is easy to find the largest match ending at index k between z and X that matches at least h strings in X. The idea is to keep track of a window in column k+1 of the prefix and divergence arrays. We will keep track of the boundaries of the window, 0≤f<g≤N. f is the index of the first haplotype in the window and g is the index of the first haplotype after f not in the window. We will also keep track of e, the starting position of the match. We start with $e=\text{min}\left(d_{z}[k+1],d_{\text{belowz}}[k+1]\right),f=t[k+1]-1$ if $d_{z}[k+1]<d_{\text{below}z}[k+1]$, otherwise f=t[k+1]. Lastly g=f+1. The number of strings in the window at any point is g-f. Now, until g-f=k, we expand the boundaries of the window to include the next longest match to z. If d[k+1][f]<d[k+1][g], then we decrement f and update e accordingly, e=max(d[k+1][f],e),f=f-1. Otherwise, we update g and e accordingly, e=max(d[k+1][g],e),g=g+1 Overall, the search of this match takes O(h) time. Figure 3 depicts this process.

### 5.2 Algorithm

Then, using similar logic to Lemma 1, we repeatedly add the longest match with h matches ending at the site just before the beginning of the last match to obtain the h-MPSC. See Lemma 2.


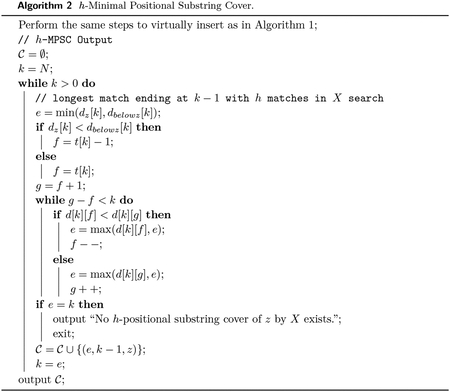


▶ **Lemma 2** (h-MPSC Modularity). *Given a subset* 𝒫 *of a* h-*minimal positional substring cover* 𝒞 *of a string* z *by* X *that covers all the indices in* {k,…,N-1} *and none of the indices in* {0,…,k-1} *for* 0<k≤N-1. *Take* m=(i,j,s), *the longest match ending at* k-1 *from* z *to* X *that is contained in* h *strings in* X. 𝒫∪{m} *is a subset of a* h-*MPSC of* z *by* X.

**Proof.** If 𝒫 is a subset of a h-minimal positional substring cover 𝒞 of a string z by X, then the (|𝒞|-|𝒫|-1)-th positional substring in 𝒞 must cover index k-1. If it didn’t, either the index k-1 is not covered by 𝒞, or another substring in 𝒞 covers index k-1. In the first case, our definition of 𝒞 is contradicted.

In the second case, if an n-th positional substring of C covers k-1, if n>|𝒞|-|𝒫|-1, our definition of 𝒫 is contradicted. If n<|𝒞|-|𝒫|-1, our definition of n-th positional substring is contradicted (n-th positional substring ends after (|𝒞|-|𝒫|-1)-th positional substring), or 𝒞 is not a minimal positional substring cover (|𝒞|-|𝒫|-1)-th positional substring starts after k-1, in which case it can be removed while maintaining the coverage of all sites).

Now, for any 𝒞, replacing the (|𝒞|-|𝒫|-1)-th positional substring with longest match ending at k-1 between z and h strings in X will yield a h-MPSC of z by X. This is because the new set is the same size as 𝒞 and all the sites 𝒞 covered are also covered by the new set. Any indices greater than k-1 are covered by 𝒫 and there are no indices less than k-1 that the original (|𝒞|-|𝒫|-1)-th positional substring covered that the longest match ending at k-1 between z and h strings in X doesn’t cover by definition. ◀

### 5.3 Time Complexity

Given a string z, and a PBWT of a set of strings X, we output a h-MPSC of z by X. Finding each positional substring takes O(h) time. Therefore, this algorithm takes O(h|𝒞|+N) where 𝒞 is the outputted cover. The N part of the time complexity comes from the virtual insertion and the h|𝒞| component from the cover search and output. See Algorithm 2 for the pseudocode of this algorithm.

## 6 Boundary cases in implementation

In the implementation of the algorithms discussed in this paper, there are boundary cases that need to be accounted for which aren’t dealt with in the pseudocode. These boundary cases are left out of the pseudocode because their handling would make the pseudocode unnecessarily long and difficult to understand. We briefly discuss these boundary cases here and how to handle them. For virtual insertion t[k] may equal M, while there is no a[k][M], this represents the fact that z would sort below every string in X in a[k]. While there is no value w[k][M][c], it is simple to calculate in constant time what this value should be. w[k][M][c] is the same as w[k][0][c+1], where c+1 is the lexicographically smallest character that is larger than c. This can also be calculated in constant time as w[k][M][c]=w[k][M-1][c]+1 if $y_{k,M-1}[k]=c$, or w[k][M][c]=w[k][M-1][c] otherwise. It should also be checked if z is longer than the strings in X, if so, no cover exists. Lastly, for the h-MPSC problem, there are two boundary cases. Firstly, if h>|X|, no cover exists. Secondly, while incrementally widening the window to contain h strings, care should be taken to avoid trying to obtain the divergence value d[k][-1] or d[k][M]. In other words, if f=0 or g=M, stop trying to increment f or decrement g respectively.

## 7 Discussion and Conclusion

In this paper, we have defined and proposed the Minimal Positional Substring Cover problem as a solution to the haplotype threading problem. We proved useful properties of Minimal Positional Substring Covers and provided a solution to the MPSC given a PBWT of X that takes time linear to the length of the query string. We also discussed variations of the Minimal Positional Substring Cover problem: leftmost, rightmost, and set maximal MPSCs. We provided solutions to these problems with the same time complexity as the original solution. Lastly, we discussed the h-MPSC problem, where every positional substring in the cover is contained in h strings in X. We provided a solution to this problem that takes O(h|𝒞|+N) time given a PBWT of X.

We have laid the ground work for the application of the Minimal Positional Substring Cover problem to haplotype threading. Immediate future work includes the modifications of the MPSC problem that would make a solution more viable for a haplotype threading solution. One such modification is an L-MPSC problem. An L-MPSC is the smallest positional substring cover of z by X that only contains positional substrings of length L or more. This is biologically useful because small matches can be considered uninformative due to the high likelihood of one occurring by random chance. Therefore, we may want to consider haplotype threading where every match is at least as long as some threshold length L. Another possibly useful variation is the o-MPSC problem. This is similar to the h-MPSC problem except instead of every substring being contained in h strings in X, every index needs to be covered by o overlapping positional substrings in C. The h-MPSC corresponds to finding a group of individuals that are closely related to each other and z for a region while the o-MPSC problem finds a group of individuals that are closely related to z for every index (they may not be closely related to each other). Lastly, a length maximal MPSC solution may be useful. A length maximal MPSC is an MPSC with a largest sum of lengths of positional substrings out of all MPSCs of z by X. A solution to this problem maximizes the overlapping regions on an MPSC, these overlaps are useful because they suggest relatedness between adjacent segments in the MPSC.

One benefit of formulating haplotype threading as a combinatorial problem like MPSC is that it enables the study of its solution space. We presented the leftmost and the rightmost solutions. All other solutions are bounded by these solutions. It may also be useful to design algorithms to derive another solution from an existing solution. E.g., given a solution 𝒞, replacing a template 𝒞[i] by another one covering the same gap between 𝒞[i-1] and 𝒞[i+1] would be another solution. Finally, it may be possible to systematically enumerate all solutions. We leave these as future work. We believe efficient solutions to these biologically useful problems exist.

## References

[1] Delaneau Olivier, Zagury Jean-François, Robinson Matthew R, Marchini Jonathan L, and Dermitzakis Emmanouil T. Accurate, scalable and integrative haplotype estimation. Nature communications, 10(1):1–10, 2019. 10.1038/s41467-019-13225-y PMC6882857 31780650

[2] Durbin Richard. Efficient haplotype matching and storage using the positional burrows–wheeler transform (pbwt). Bioinformatics, 30(9):1266–1272, 2014. 24413527 10.1093/bioinformatics/btu014 PMC3998136

[3] Li Na and Stephens Matthew. Modeling linkage disequilibrium and identifying recombination hotspots using single-nucleotide polymorphism data. Genetics, 165(4):2213–2233, 2003. 14704198 10.1093/genetics/165.4.2213 PMC1462870

[4] Loh Po-Ru, Danecek Petr, Palamara Pier Francesco, Fuchsberger Christian, Reshef Yakir A, Finucane Hilary K, Schoenherr Sebastian, Forer Lukas, McCarthy Shane, Abecasis Goncalo R, Reference-based phasing using the haplotype reference consortium panel. Nature genetics, 48(11):1443–1448, 2016. 27694958 10.1038/ng.3679 PMC5096458

[5] Lunter Gerton. Haplotype matching in large cohorts using the li and stephens model. Bioinformatics, 35(5):798–806, 2019. 30165547 10.1093/bioinformatics/bty735 PMC6394399

[6] Naseri Ardalan, Holzhauser Erwin, Zhi Degui, and Zhang Shaojie. Efficient haplotype matching between a query and a panel for genealogical search. Bioinformatics, 35(14):i233–i241, 2019. 31510689 10.1093/bioinformatics/btz347 PMC6612857

[7] Naseri Ardalan, Liu Xiaoming, Tang Kecong, Zhang Shaojie, and Zhi Degui. Rapid: ultra-fast, powerful, and accurate detection of segments identical by descent (IBD) in biobank-scale cohorts. Genome biology, 20(1):1–15, 2019. 31345249 10.1186/s13059-019-1754-8 PMC6659282

[8] Naseri Ardalan, Zhi Degui, and Zhang Shaojie. Multi-allelic positional Burrows-Wheeler transform. BMC bioinformatics, 20(11):1–8, 2019. 31167638 10.1186/s12859-019-2821-6 PMC6551244

[9] Rubinacci Simone, Delaneau Olivier, and Marchini Jonathan. Genotype imputation using the positional burrows wheeler transform. PLoS genetics, 16(11):e1009049, 2020. 33196638 10.1371/journal.pgen.1009049 PMC7704051

[10] Sanaullah Ahsan, Zhi Degui, and Zhang Shaojie. d-PBWT: dynamic positional Burrows-Wheeler transform. Bioinformatics, 37(16):2390–2397, 2021. 33624749 10.1093/bioinformatics/btab117 PMC12158185

[11] Yue William, Naseri Ardalan, Wang Victor, Shakya Pramesh, Zhang Shaojie, and Zhi Degui. P-smoother: Efficient PBWT smoothing of large haplotype panels. Bioinformatics Advances, 2022. doi:10.1093/bioadv/vbac045. PMC9245627 35785021

